# What is new in the treatment of eosinophilic eosophagitis?

**DOI:** 10.1186/2045-7022-1-S1-S69

**Published:** 2011-08-12

**Authors:** Amal Assa'ad

**Affiliations:** 1 Cincinnati Children's Hospital Medical Center, Division of Allergy & Immunology, Cincinnati, USA

## 

In November 2009, in the beautiful city of Venice, Italy, at the first EAACI Pediatric Allergy and Asthma Meeting, I discussed Eosinophilic Esophagitis (EoE) as an emerging disorder. I discussed the diagnosis of the disorder based on histopathologic findings of esophageal eosinophilia of >15 cells per high power field, after 3 months of treatment with a proton pump inhibitors. I discussed several therapeutic modalities that are used in practice, some with good evidence, others without: dietary interventions: elimination diets, six food elimination diet and elemental diets; medical management: topically administered steroids, aerosolized fluticasone or budesonide; and experimental therapies: anti-IL5 and anti-IgE antibodies. Now in 2011, I am pleased to return to the beautiful city of Venice to present at the first EAACI Food Allergy and Anaphylaxis Meeting (FAAM) on exciting new advances in the treatment of EoE. The advances are a translation from the bench to the bedside of much work on the pathogenesis and genetics of EoE. I am also pleased to see, that while reports of EoE had been mostly from the USA and Switzerland, and mostly on pediatric patients, there is ample literature now from several additional European countries and emerging expertise on adult patients. I will be presenting new data on the use of topical budesonide both in pediatrics and adults, the continuing debate on the effect of proton pump inhibitors in differentiating EoE from reflux eosophagitis and in treatment of EoE. Several studies with biologic agents, anti-IL 5 antibodies, mepolizumab and reslizumab, have been completed and yielded interesting data on the disorder, its course and its response to treatment. Finally, I will present data on novel mechanisms of EoE, from mast cell and B cell involvement and local IgE production to a role of IL-13. The recently elucidated role of IL-13 in EoE has resulted in an ongoing clinical trial of anti-IL-13 antibody in patients with EoE. I am looking forward to hearing from you, FAAM attendees on your own experiences with patients with EoE and your innovation in further elucidating the triggers for EoE and the successes of your management.

